# Psychological disorders among Spanish Nursing students three months after COVID‐19 lockdown: A cross‐sectional study

**DOI:** 10.1111/inm.13086

**Published:** 2022-11-03

**Authors:** Itziar Mendez‐Pinto, Maria Antuña‐Casal, Maria‐Pilar Mosteiro‐Diaz

**Affiliations:** ^1^ Postgraduate International Center University of Oviedo Oviedo Spain

**Keywords:** COVID‐19, mental health, Nursing students, pandemic

## Abstract

The COVID‐19 pandemic had a notable impact on the psychological well‐being of a large part of the population, putting them at risk of developing depressive symptoms, different levels of anxiety disorders, and posttraumatic stress disorder (PTSD). One group considered to be at high risk are Nursing students; they were affected as learning strategies changed and clinical practices were cancelled. This study attempts to assess the psychological impact COVID‐19 pandemic had on Nursing students and to explore the sociodemographic differences that can be risk factors for mental health disturbance. The psychological impact was evaluated using the Impact of Event Scale‐Revised (IES‐R) and Hospital Anxiety and Depression Scale (HADS). The study took place 4 months after the state of alarm was declared over in Spain. From a total sample of 304 Nursing students, 26.7%, 39.8%, and 15.5% showed PTSD, anxiety, and depression symptoms, respectively. Severe levels of psychological impact have been associated with being a female, a smoker, and feeling fear and stress. Having a relative test positive has been linked to lower anxiety levels while being afraid or stressed to higher anxiety levels. Being a female, co‐habit with friends and feeling stress have been associated with higher depression levels.

## BACKGROUND

The emergence of SARS‐CoV‐2 was first observed during early December of 2019 when unexplained pneumonia cases appeared in the Chinese city of Wuhan (Mavragani 2020).

Since then, the high virus transmission rate enhanced its rapid spread around China and other countries worldwide (Lai *et al*. 2020). On 11 March 2020, the scale of the crisis resulted in the World Health Organization (WHO) declaring a pandemic; and reached the toll of one million COVID‐19 cases worldwide by 4 April 2020 (World Health Organization 2021). Since May 2021, the WHO had identified various mutations affecting the virus, some of which resulted in advantages such as increased transmissibility. The variants Alpha, Beta, Gamma, Delta, and Omicron have all been identified as variants of concern due to their increased transmissibility and reduced vaccine efficacy (Mavragani 2020; World Health Organization  2022).

The high transmission rate, morbidity, and mortality of the virus forced some countries to develop strict isolation measures. Firstly, to minimize the spread of the virus, and secondly, to prevent national health systems from collapsing (Mavragani 2020). In Spain, on 14 March 2020, the government, supported by the Royal decree 463/2020, declared a “State of alarm” for the management of the COVID‐19 crisis resulting in the confinement of the population of all the national territory of Spain until 9 May of 2021 (Spanish Ministry of Health 2020).

COVID‐19 was a significant health challenge worldwide. It had a serious impact on the health of a large part of the population, not only the risk of contracting a viral infection but also the negative impact on the mental health (Yıldız 2021).

The pandemic changed every aspect of our personal lives, employment, social activities, education, and community connection; causing anxiety, fear, hopelessness, despair, loss of purpose, insecurity, and a specific intolerance to uncertainty among the general population (Hamadeh Kerbage *et al*. 2021; Reverté‐Villarroya *et al*. 2021; Usher *et al*. 2020). Previous studies have designated COVID‐19 as a remarkable stressor that can cause physiological problems (Zhang *et al*. 2021a, 2021b). Individuals with no prior mental health history can also develop depressive symptoms, different levels of anxiety disorders, and PTSD (Göl & Erkin 2021).

The COVID‐19 pandemic has disrupted many aspects of global health, economy, and politics, but also education (Laranjeira *et al*. 2021). The pandemic significantly impacted university students from different countries; according to UNESCO, 60% of students worldwide have been affected by national lockdowns (Patelarou *et al*. 2021). Nursing Faculties globally modified the teaching approach to a virtual format and forced the cancellation of educational activities (Reverté‐Villarroya *et al*. 2021; Usher *et al*. 2020). Consequently, clinical practices were interrupted primarily due to fear of spreading and uncertain knowledge of the disease (Reverté‐Villarroya *et al*. 2021). The impact COVID‐19 had on Nursing students was obvious. Some were concerned with the long‐term impact on their future careers, late graduation, or facing changes of the global recession caused by COVID‐19 pandemic (Patelarou *et al*. 2021), whilst other students had been in contact with the virus or even suffered from the disease. Some fourth‐year Nursing students volunteered to provide healthcare for vocational and moral reasons, entering the world of work without fully finishing their clinical practicum and putting their health at risk (Roca *et al*. 2021). In Spain, due to the lack of healthcare workers and the overburdened Spanish health system, the Spanish authorities regulated the contracting of final‐year health sciences students (Patelarou *et al*. 2021; Reverté‐Villarroya *et al*. 2021). In this respect, numerous Nursing students faced the dichotomy of choosing between their safety and pursuing a Nursing career (Usher *et al*. 2020).

COVID‐19 appeared as a new stressor for Nursing students (Yıldız 2021), repercussing directly on students’ stress, anxiety, and depression levels (Marcén‐Román *et al*. 2021). A study by Reverté‐Villarroya *et al*. (2021) comparing mental health problems between Spanish Nursing students in 2017 prior to COVID‐19 and in 2020 during the pandemic established a two‐times greater risk of nurses suffering mental health problems for those studying during the COVID‐19 crisis. A different study conducted in Turkey in June 2020 indicated that 71.5% of Turkish Nursing students were at risk of developing mental health problems (Göl & Erkin 2021).

Depression, anxiety, and stress are the most common psychiatric disorders among University students (Cheung *et al*. 2016). A study performed in Spain to measure the stress perceived by University Health students after 1 year of the pandemic indicates a 71.4% of participants had anxiety symptoms and 81% had depression symptoms (Marcén‐Román *et al*. 2021). Furthermore, according to a comparative study about depression in Nursing students during COVID‐19, Spanish students (59.1%) experienced more depression symptoms than Greek (21.8%) or Albanian (34.5%) students. Demonstrating that demography is a strong predictor of developing depression, which can be attributed to the increased public concern and anxiety due to the rise of deaths in the area (Patelarou *et al*. 2021).

PTSD is one more negative impact of COVID‐19, as its prevalence has been raised in the general population (Shevlin *et al*. 2020). PTSD is well known to be developed as a response to a direct or indirect exposure to a traumatic event (Zhang *et al*. 2021a, 2021b).

A higher prevalence of PTSD was found during COVID‐19 among students, and having a friend or family member as a healthcare worker was also found to increase the likelihood of developing PTSD (Sultana *et al*. 2021). Healthcare workers are more exposed to PTSD than the general population and are the first to face the disease due to their patient‐facing role (Bassi *et al*. 2021).

A study in Turkey in May 2020 concluded that 34.5% of University students met the criteria for PTSD during the pandemic (Cam *et al*. 2022). In comparison, a study in Bangladesh showed that 40.91% of University students suffered from PTSD during home confinement (Sultana *et al*. 2021).

Most of the studies in our literature review were carried out during the national lockdowns. Not many articles were published concerning the psychological impact of Nursing students after the confinement period was over. Therefore, the aims of this research were: (a) to know the prevalence of PTSD, anxiety and depression among Nursing students, (b) to identify factors that contribute to a higher psychological impact by comparing the demographic and social characteristics of the sample as possible predictors, and (c) to identify a student's profile with a higher risk of psychological impact.

## METHODS (DESIGN, DATA COLLECTION AND ANALYSIS)

### Study design

The study adopted a descriptive, quantitative, and cross‐sectional survey design. This study was conducted at two Nursing Faculties in the Principality of Asturias (Northern Spain). To ensure the quality of the research, we used The Strengthening the Reporting of Observational Studies in Epidemiology (STROBE) checklist for cross‐sectional studies.

### Participants and data collection

The target population of this study consisted of undergraduate Nursing students affected by the outbreak of the COVID‐19 pandemic during the development of their bachelor's degree. Regarding the sample size, our population were the Nursing students who attended lectures on the day and time previously decided by the research team. Before the data collection, we estimated the minimum sample required for the study, *n* = 165. This was done by the estimated proportion where the total population (*N*) was 500 students, Za 2 = 1.962 (confidence level (1−*α*) = 95%), statistical significance (*P*) =0.05, (*q*) = 1–*P* (1–0.05 = 0.95), and precision (*d*) = 3%. Finally, the total size of participants was *n* = 304.

The inclusion criteria in the study were as follows: (a) being undergraduate Nursing students enrolled in one of the faculties; (b) had been affected by the pandemic outbreak while studying; (c) attending lectures on the date of the data collection; (d) willing to participate in the study; and (e) those who fully completed the questionnaires. On the other hand, our exclusion criteria were the first‐year students who were not affected by the pandemic while studying Nursing.

The study took place during the first semester of the academic year 2021–2022. Data collection started on 10 September 2021, coinciding with the beginning of lectures and tutorials at University, and it was finally completed by 12 November. The time setting of the study corresponded with the end of the State of Alarm (9 May 2021) by the Spanish Ministry of Health, 4 months prior to the data gathering.

Regarding the setting, recruitment of participants and data collection took place on the same date. Researchers firstly explained the study and questionnaire to the students; those who gave consent to participate were given the self‐reported questionnaires. The research was designed to minimize observational bias.

### Measurements

Our research was conducted face‐to‐face via paper‐based questionnaires during the lectures of the Nursing Faculties. The questionnaires consisted of three sections: student information, psychological impact scale, and anxiety and depression scale. All questionnaires have previously been translated and validated in the Spanish language.

1. Sociodemographic variables (Age, Gender, Marital status, Faculty, Academic year, Coexistence, Children, Dependents, Smoking habit, and Place of residence), Labour variables (Employment situation) and variables related to the personal COVID‐19 situation (COVID‐19 status, Family member positive, Friend positive, Fear, Stress, and Vaccination status)
2. Impact of Event Scale‐Revised (IES‐R): The study uses the Impact of Event Scale‐Revised (IES‐R) of the Spanish version validated by Báguena *et al*. (2001). This self‐reported scale is a revision designed by Weiss & Marmar in 1997. The questionnaire consists of 22 items to measure the subjective response to a specific traumatic event (Joseph 2000). This scale is not used to diagnose PTSD but to capture the criteria. Items are scored on a five‐option scale from 0 (not at all) to 4 (extremely). The IES‐R includes a total score (ranging from 0 to 88) and three different subscales that aim to reflect the symptoms of intrusion (8 items), avoidance (8 items), and hyperarousal (6 items) (Cam *et al*. 2022; Weiss 2007). Higher scores reflect a higher distress level of the person taking the test. The total IES‐R scores were divided into normal (0–23), mild psychological impact (24–32), moderate psychological impact (33–36), and severe psychological impact (>37) (Weiss 2007). The internal consistency of the IES‐R was high with a Cronbach's alpha of 0.95 (Báguena *et al*. 2001).
3. Hospital Anxiety and Depression Scale (HDAS): The Hospital Anxiety and Depression Scale (HADS) was designed as a screening instrument for anxiety and depression in a hospital setting by Zigmond and Snaith (1983). Even though it was first intended for a hospital setting, the tool has been acceptable for different contexts such as community or higher education (Pais‐Ribeiro *et al*. 2007). This scale contains 14 items with multiple choice questions, composed of two subscales referred to as anxiety (HADS‐A) and depression (HADS‐D), formed of seven items. Scores for each item have four different answers ranging from zero to three, the total range scores from 0 to 21. Students who score 0–7 are considered normal, 8–14 borderline, and 15–21 elevated. Therefore, from 8 to equal of the maximum (21), students will be deemed to have anxiety and/or depression symptoms; however, this is not an official diagnosis. To interpret the results, the higher a participant score, the greatest chances the person has to develop an anxiety or depression disorder (Pais‐Ribeiro *et al*. 2007). This scale is brief and easy to understand. The study uses the HADS on the Spanish version validated by Herrero *et al*. (2003). The internal consistency for this validation was found to be acceptable, with a Cronbach's alpha of 0.90 for the HADS tool, 0.85 for the anxiety subscale, and 0.84 for the depression subscale (Herrero *et al*. 2003).

### Data analysis

Data were processed and analysed in Statistical Package for the Social Science version 27.0 (IBM‐SPSS v 27) software. The sample characteristics were analysed and described by descriptive statistics, such as mean, median, minimum, maximum, or measures of dispersion such as the standard deviation to quantitative type and percentages variables. Sociodemographic characteristics of the sample were identified as the independent variables, while the IES‐R score, HADS‐Anxiety score, and HADS‐Depression score were the study's dependent variables.

The normality of distribution of the continuous variables was analysed using the Kolmogorov–Smirnov test and graphics as histograms and Q–Q plots. Identifying that the continuous variables followed a normal distribution. In order to show the correlation of the quantitative variables, the Spearman's Rho test was applied. The significance level of 0.05 was applied. No correlation was found between the independent variables of age and scores (IES‐R and HADS). The qualitative variables were expressed as absolute and relative frequencies, using the bivariate analysis of chi‐square. The statistical tests of ANOVA and Student *t*‐test were used to examine statistically significant differences in the mean of IES‐R and HADS concerning the quantitative independent variable of age. The level of significance for acceptance was determined to be *P* < 0.05.

Regarding the treatment of missing data, those participants who did not answer more than two items of the IES or HADS were excluded from the study. To replace missing values, we used a linear trend.

## RESULTS

### Demographic characteristics of participants

This cross‐sectional study included a total sample of 304 Nursing students. Among those students, 157 (51.6%) were from the Nursing Faculty of Oviedo, while 147 (48.4%) were from the Nursing Faculty of Gijón. The mean age was 22.11 (SD ± 4.905) years. The majority of responders were female (87.5%), single (89.1%), with no children (95.7%), living with parents/grandparents (84.2%), unemployed full‐time students (85.9%), non‐smokers (89.8%), and living in an urban area (78%). The distribution of participants for 2nd, 3rd, and 4th year students was 32.6%, 33.2%, and 34.2%, respectively.

Regarding the infectious status, only 18.1% of the participants had been infected with SARS‐CoV‐2; this percentage increases when referring to infected relatives (45.7%) and friends (81.9%). When the participants were asked if they were afraid, we could see the majority (80.6%) were not, when asked about stress, 60.9% stated they were feeling stressed. From this study, we observed that 304 (100%) of the participants have been vaccinated for the COVID‐19 virus. The sociodemographic characteristics are presented in Table 1.

**Table 1:** Demographic characteristics of participants

Characteristics	N or mean ± SD	%
Age (year)	22.11 ± 4.905	
Gender		
Male	38	12.5
Female	266	87.5
Faculty		
Oviedo	157	51.6
Gijón	147	48.4
Academic Year		
2nd year	99	32.6
3rd year	101	33.2
4th year	104	34.2
Marital status		
Single	271	81.9
Live‐in couple/Married	33	10.9
Divorced/Separated/Widow	0	0
Coexistence		
Living with Parents/Grandparents	256	84.2
Alone	13	4.3
With friends	11	3.6
Another situation	24	7.9
Children		
Yes	13	4.2
One Child	8	2.6
Two Children	5	1.6
No	291	95.7
Dependents		
No	291	95.7
Yes	13	4.3
Smoking habit		
Non‐Smokers	273	89.8
Smoker		10.2
Employment situation		
Employed	27	8.9
Unemployed	16	5.3
Full‐time student	261	85.9
Place of residence		
Rural area	67	10.9
Urban area	237	89.1
Were you COVID‐19 positive?		
No	249	81.9
Yes	55	18.1
Did any of your family members suffer from COVID‐19?		
No	165	54.3
Yes	139	45.7
Did any of your friends suffer from COVID‐19?		
No	33	10.9
Yes	271	89.1
Are you afraid?		
No	245	80.6
Yes	59	19.4
Do you feel stressed?		
No	119	39.1
Yes	185	60.9
Are you vaccinated?		
No	0	0
Yes	304	100

### Psychological impact (IES‐R)

The overall mean score of the IES‐R for the participants was 21.24 (SD ± 15.409) (min: 0; Max: 63). This result indicates that 55 (18.1%) of respondents were severely impacted, 26 (8.6%) had a moderate psychological impact, 41 (13.5%) had mild, and 182 (59.9%) were within the normal parameters of the measurement tool. According to this result, the prevalence of PTSD symptoms is 26.7%. For the IES‐R levels within the three subscales of this questionnaire, we obtained the following scores (Mean): Avoidance (8.63), Hyperarousal (6.03), and Intrusion (6.58).

### Anxiety and depression (HADS)

The average score for anxiety and depression was 7.02 (SD ± 3.893) and 4.01 (SD ± 3.265).

A normal level on the HADS‐Anxiety was found in 60.2% of participants, 35.5% had borderline levels, and 4.3% elevated levels. Regarding HADS‐Depression, 84.5% of the sample scored normal, 14.5% borderline, and 1.0% elevated.

### Correlation analysis

#### Sociodemographic variables related to IES‐R levels

Chi‐square was used to analyse the correlation between the sociodemographic variables and the IES‐R levels. Four of these independent variables were significantly correlated with the IES‐R: Gender, Smoking habits, Fear, and Stress.

From a total sample of 304 Nursing students, we observed a severe psychological impact of 19.5% in females compared with 7.8% in males. The severe psychological impact is higher in smokers (22.5%) than in non‐smokers (17.5%). Regarding the questions about fear and stress, those participants who stated “yes” for the questions had an IES‐R severe prevalence of 37.2% for fear and 23.7% for stress; compared to 13.4% and 9.2% correlated for those whose answer was “no” (Table 2).

**Table 2:** Correlations between IES‐R levels and sociodemographic variables

	Levels of IES‐R					
	Normal	Mild	Moderate	Severe	Total	*P* *
Gender						
Female	159	36	19	52	266	0.059
Male	23	5	7	3	38	
Total	182	41	26	55	304	
Smoking Habits						
Non‐Smoker	171	33	21	48	273	0.044
Smoker	11	8	5	7	31	
Total	182	41	26	55	304	
Fear						
Yes	20	9	8	22	59	<0.001
No	162	32	18	33	245	
Total	182	41	26	55	304	
Stress						
Yes	92	27	22	44	185	<0.001
No	90	27	4	11	119	
Total	182	41	26	55	304	

* Chi‐square test.

#### Sociodemographic variables related to HADS‐ anxiety levels

In a total sample of 304 Nursing students, 35.5% revealed borderline anxiety levels, and 4.2% had severe anxiety levels. The anxiety was found to be significantly associated with fear, stress, and a relative who tested positive for COVID‐19. Having a relative infected with the COVID‐19 virus was associated with lower anxiety levels (*P* = 0.009). Also, reporting fear or stress was associated with having higher anxiety levels with *P* = <0.001 for both (Table 3).

**Table 3:** Correlation between HADS‐anxiety levels and sociodemographic variables

	Levels of HADS‐anxiety				
	Normal	Borderline	Elevated	Total	*P* *
Relative tested positive					
Yes	92	46	1	139	0.009
No	91	62	12	165	
Total	183	108	13	304	
Fear					
Yes	23	31	5	59	<0.001
No	160	77	8	245	
Total	183	108	13	304	
Stress					
Yes	83	89	13	185	<0.001
No	100	19	0	119	
Total	183	108	13	304	

* Chi‐square test.

#### Sociodemographic variables related to HADS‐Depression levels

The prevalence of depression was higher among females living with friends and reporting stress. Three sociodemographic variables (gender, coexistence, and feeling stress) were identified as having a statistically significant association with depression levels.

In a total sample of 266 females and 38 males, depression was only observed in females, with a prevalence of 16.5% for borderline and 1.1% for elevated (Table 4).

**Table 4:** Correlations between HADS‐depression levels and Sociodemographic variables

	Levels of HADS‐depression				
	Normal	Borderline	Elevated	Total	*P* *
Gender					
Female	219	44	3	266	0.019
Male	38	0	0	38	
Total	257	44	3	304	
Coexistence					
Parents/Grandparents	222	31	3	256	0.040
Alone	12	1	0	13	
Friends	7	4	0	11	
Other	16	8	0	24	
Total	257	44	3	304	
Stress					
Yes	146	36	3	185	0.003
No	111	8	0	119	
Total	257	44	3	304	

* Chi‐square test.

Co‐habitation was associated with depression levels. Students living with their parents or grandparents had a prevalence of 1.1% for elevated levels, while those living with friends, alone, or in another situation was 0%. For the borderline level, the higher prevalence was those living with friends (36.3%), followed by those living in another situation (33.3%), living with their parents (12.1%), and living alone (7.6%).

Perception of stress has been correlated to depression levels as well. Those students who perceived stress (21% *vs* 6.7%; *P* = <0.001) were more likely to develop depression symptoms.

## DISCUSSION

Psychopathology such as PTSD, anxiety, and depression are commonly observed during pandemics and there is enough evidence to associate them with the COVID‐19 pandemic (Gao *et al*. 2021; Keskin & Özkan 2021). At the time of the study, there was not much literature regarding the effects on mental health post‐pandemic.

The research results indicate a significant prevalence of PTSD, anxiety, and depression symptoms among Nursing students, 26.7%, 39.8%, and 15.5%, respectively.

In our study, many Nursing students reported PTSD symptoms; a total of 26.7% scored an IES‐R of 33 or higher; 13.5% were mild, 8.6% were moderate, and 18.1% were severe, according to IES‐R measurement. This prevalence is lower than other studies, such as the one developed by Gao *et al*. (2021) which demonstrated a prevalence of 44.5% among Chinese college nurses; or 34.5% for Turkish university students (Cam *et al*. 2022), or the Japanese study which showed a prevalence of 58.5% for PTSD symptoms (Tanji & Kodama 2021) and the Bangladeshi study that showed 40.9% of prevalence for PTSD symptoms in university students (Sultana *et al*. 2021). Hence, the PTSD prevalence we obtained is higher than a study developed in China 1 month after the pandemic started, with a prevalence of 2.7% (Tang *et al*. 2020). Yet, the prevalence of severe cases in our study (18.1%) is higher if we compare it with the prevalence of other studies, such as 12.5% of University students from Valladolid in Spain (Odriozola‐González *et al*. 2020) and 12.5% Nursing students from Japan (Tanji & Kodama 2021).

According to the literature, Nursing students already have significant stress levels due to their education. Additional risk factors from the pandemic will increase the prevalence of anxiety these students experience (Kuru Alici & Ozturk Copur 2022). In our study, 39.8% of participants showed anxiety symptoms. Among them, 35.5% were borderline, and 4.3% were elevated. This is lower than other studies developed during the lockdown period, as 42.8% among Israeli Nursing students (Savitsky *et al*. 2020); 55.0% among Chinese Nursing students (Zhu *et al*. 2021); 51.5% for severe symptoms in Turkish Nursing students (Kuru Alici & Ozturk Copur 2022); or 48.6% of Turkish university students (Cam *et al*. 2022).

As regards depression, the prevalence of this study was 15.5%, in which, 14.5% were borderline, and 1.0% were elevated. A study performed during the COVID‐19 outbreak on the general population of Spain where 18.7% of the sampled population demonstrated depression symptoms (González‐Sanguino *et al*. 2020). Even so, other studies designed with university students during the pandemic in Bangladesh (Sultana *et al*. 2021), Turkey (Cam *et al*. 2022), Nursing students in Albania (Mechili *et al*. 2021), and Nursing students in China (Zhu *et al*. 2021) showed a prevalence of depression of 52.8%, 64.6%, 25.2%, and 56.4%, respectively. In contrast, studies on Nursing students in China had a lower prevalence of 2.9% (Gao *et al*. 2021) and 9.0% (Tang *et al*. 2020). While a study developed 1 year after the pandemic in January 2021 among health science students from Spain showed that 81% of the sample had depression symptoms (Marcén‐Román *et al*. 2021). These differences may be explained by the differing phases of the pandemic when the data collection was performed. Previous studies have demonstrated that stress levels increase significantly when comparing periods of before and after restrictions (Keskin & Özkan 2021).

### Co‐relations of sociodemographic variables and psychological impact, anxiety, and depression

There is enough evidence demonstrating the correlation between age and psychological impact, sufficiently so that age is an important predictor of psychological impact (Carmassi *et al*. 2020; González‐Sanguino *et al*. 2020; Kuru Alici & Ozturk Copur 2022; Picaza Gorrochategi *et al*. 2020; Patelarou *et al*. 2021). Despite that, our study could not find a significant correspondence between the parameters of psychological impact, anxiety, and depression, with the participants' age.

In previous studies, psychological impact levels among female students were frequently higher than in male students (Cam *et al*. 2022; da Silva *et al*. 2021; González‐Sanguino *et al*. 2020; Hasuike *et al*. 2021; Kuru Alici & Ozturk Copur 2022; Marcén‐Román *et al*. 2021; Savitsky *et al*. 2020; Sultana *et al*. 2021; Tang *et al*. 2020). The risk of developing PTSD after experiencing a traumatic event is twice as high for females as it is for males (Cam *et al*. 2022). Previous research found a link between university students enrolling in health‐related courses and female students who are more depressed (da Silva *et al*. 2021), matching with the findings of this study. Gender has been demonstrated as a solid predictor to develop PTSD and depression symptoms during the COVID‐19 pandemic. It is important to note that females constitute the majority of our Nursing student population (87.5%), which can explain the higher prevalence of PTSD and depression.

Diverse studies found working during COVID‐19 (da Silva *et al*. 2021; Marcén‐Román *et al*. 2021), parental status (Savitsky *et al*. 2020; Zhu *et al*. 2021), COVID‐19 infectious status (Sultana *et al*. 2021), or the academic year (Kuru Alici & Ozturk Copur 2022) as predictors for a negative impact on students’ mental health. On the contrary, our study did not find any statistically significant relation for these parameters.

In the case of co‐habitation, it has been demonstrated that as the number of people living together at home increases, the level of depression decreases (Keskin & Özkan 2021). This relates to the results of our study where we found a statistically significant correlation between co‐habitation and depression; those students living with parents/grandparent (12.1%) and alone (7.6%) experiences a lower prevalence of depression than the ones living with friends (36.3%) or another situation (33.3%). Some explanations for this phenomenon are that students living alone have less fear of infecting their loved ones, as living with high‐risk groups has been linked with increased levels of depression in previous studies (Laranjeira *et al*. 2021). On the other hand, feelings like loneliness and a lack of social connection were the cause of psychological discomfort during the pandemic (da Silva *et al*. 2021; De Micheli *et al*. 2021). We believe that those students living with their families felt more supported.

Our findings contributed to the correlation between fear of infection and increased PTSD (Sultana *et al*. 2021) and anxiety symptoms (Savitsky *et al*. 2020). In our study, those participants who reported being afraid had higher levels of PTSD (37.2%) and anxiety (60.9%) than those who stated not being afraid.

Regarding perceived stress, there is a relationship between anxiety and depression (Marcén‐Román *et al*. 2021). When stress is under control, an adequate level can improve the body's resistance and function as a protective mechanism. When out‐of‐control, or excessive, stress has a harmful effect on the autonomic nervous system and cortex, resulting in physical and psychological symptoms. The COVID‐19 pandemic and associated socio‐economic changes are stressful and reduce effective coping techniques such as hope and life satisfaction (Laranjeira *et al*. 2021). Our research found a link between perceived stress and anxiety, depression, and psychological impact.

The literature shows a significant positive relationship between anxiety and a COVID‐19 positive relative (Kuru Alici & Ozturk Copur 2022; Zhang *et al*. 2021a, 2021b). On the contrary, not having a relative infected was demonstrated to be a predictor for anxiety in our study. Those students who had a relative already infected had a lower prevalence of anxiety than those who did not (35.9% *vs* 44.7%). According to the results of Marcén‐Román *et al*. (2021) those students who had less contact with COVID‐19 had more stress, and were more likely to suffer from anxiety and depression.

Results indicate an 18.1% prevalence of infected participants in our study. While the majority (89.1%) of participants had a friend who got infected with COVID‐19, and 45.7% a relative tested positive in our study.

Various studies demonstrate that the place of residence is a strong predictor of psychological impact. The prevalence of psychological distress is higher in locations where COVID‐19 is prevalent, during high‐risk seasons and the difference in legislation (Gao *et al*. 2021). In our case, Spain has been affected badly by the virus, as living in Spain has proved to be a predictor of psychological impact (Patelarou *et al*. 2021). Nevertheless, the moment our data collection took place was not a high‐risk period, the population was more relaxed after the end of the lockdown measurements. Previous studies also found a difference in the psychological impact between students living in a rural or urban areas, demonstrating that living in rural areas is a protective factor against anxiety during the COVID‐19 outbreak (Kuru Alici & Ozturk Copur 2022); however, our study could not find a relation.

Finally, students with outstanding fear scores were more prone to engage in unhealthy behaviours like smoking (Tavolacci *et al*. 2021). Furthermore, people who use mental health services are more likely to smoke (Peckham *et al*. 2021). Of all participants, 22.5% who had PTSD disorder were smokers. As a result, smoking was a predictor of developing PTSD symptoms in this research; concurring with the results obtained in a study in Bangladesh that smoking habits increase students' mental health disparity (Alam *et al*. 2021).

A limitation to keep in mind is the difference in the psychological impact that we can observe across the literature. These disparities can be attributed to various factors such as the data collection period, cultural differences, the period of the pandemic, government measurements, and the healthcare system responses. Additionally, other study's limitations are the cross‐sectional design and self‐reported measures. A cross‐sectional approach limits the causal‐effect connections between variables. We want to highlight the necessity of future longitudinal research.

## CONCLUSIONS

When it comes to understanding the psychological impact of the COVID‐19 pandemic among Nursing students, this research added new pieces of evidence and perspectives. Nursing students from our study presented a notable psychological impact due to the COVID‐19 pandemic, which can still be appreciated after the relaxation of COVID‐19 restrictions. We believe the outcome of the psychological distress results from the exceptional situation of COVID‐19.

Given the above, our research has identified various predictors of psychological distress for Nursing students. Gender, smoking habit, coexistence, not having a relative tested positive, perceived stress, and fear were risk predictors for a more significant psychological impact. Predictors including being female, a smoker, feeling fear, and stress have been linked to a higher risk for PTSD. Feeling fear or stress and not having a relative tested positive have also been identify as risk predictors of anxiety; while being female, living with friends and feeling stress increased the likelihood of depression.

According to our results, a student profile with a higher risk would be a female smoker, living with friends who is feeling fear and stress.

### Relevance for clinical practice

We suggest more visibility for the University's psychological support services, developing counselling services, assistance, and promoting programs to help the psychological well‐being of the university students. The results of this study could be used to identify the profile of students at risk of developing mental health problems and undertake appropriate intervention, especially for those students who are at a greater risk of developing mental issues such as Nursing students. A follow‐up assessment is needed to evaluate the symptoms and assess the long‐term consequences during and after the pandemic. As the future of this COVID‐19 pandemic remains unpredictable, further studies need to focus on suggesting assessing methods and coping strategies.

## Funding statement

This research did not receive any financial support.

## ETHICAL APPROVAL

The study was designed according to the Code of Ethics of the World Medical Association for legal and ethical aspects (Declaration of Helsinki). Before beginning the research, permission to conduct the study was obtained from the Ethics Research Committee of the Principality of Asturias (2020.116). All data collected was anonymized to ensure the privacy of the participants.

## Data Availability

The data that support the findings of this study are available from the corresponding author upon reasonable request.
